# Risk factors and predictive model for early surgical site infection following single-level PLIF in diabetic patients

**DOI:** 10.3389/fsurg.2025.1709831

**Published:** 2025-12-18

**Authors:** Xusheng Li, Ahmad Nazrun Shuid, Mohd Fairudz Mohd Miswan, Donghui Cao, Xiao Zhang, Yanrong Tian, Haifeng Yuan

**Affiliations:** 1 Department of Spinal Orthopaedics, General Hospital of Ningxia Medical University, Yinchuan, China; 2 Faculty of Medicine, Universiti Teknologi MARA, Jalan Hospital, Sungai Buloh, Selangor, Malaysia

**Keywords:** early surgical site infection, lumbar interbody fusion, prediction model, risk factors, serum biomarkers

## Abstract

**Objective:**

This study aims to investigate the predictive value of postoperative serum biomarkers for early surgical site infection (SSI) following single-level posterior lumbar interbody fusion (PLIF) in diabetic patients, and to construct an infection risk prediction model based on key indicators. The goal is to provide a theoretical basis and tool support for precise clinical prevention and control.

**Methods:**

A retrospective analysis was conducted on 1,680 diabetic patients who underwent single-level PLIF in our Hospital, from January 2011 to December 2024. Among these, 165 patients developed early SSI. Univariate analysis was performed using–Whitney *U*-test and the chi-square test. Subsequently, LASSO regression was employed for variable selection and dimensionality reduction, and independent risk factors were determined using multivariate logistic regression. Data were divided into training and validation sets in a 7:3 ratio, and a prediction model was constructed using 10-fold cross-validation. The model's predictive performance and clinical utility were comprehensively evaluated with calibration curves, receiver operating characteristic (ROC) curves, and decision curve analysis (DCA).

**Results:**

Univariate analysis revealed that patients in the infection group had significantly higher postoperative day 3 fasting blood glucose (FPG pod3: 17.18 vs. 14.13 mmol/L), C-reactive protein (CRP pod3: 281.70 vs. 111.17 mg/L), white blood cell count (WBC pod3: 41.28 vs. 16.90 × 10^9^/L), and 4 other postoperative inflammatory markers compared to the non-infection group (all *P* < 0.001). Multivariate logistic regression further identified CRP pod3 (OR = 1.025, 95% CI: 1.01–1.04, *P* < 0.001), WBC pod3 (OR = 1.27, 95% CI: 1.17–1.43, *P* < 0.001), Erythrocyte Sedimentation Rate (ESR) pod3 (mm/h) (OR = 1.021, 95% CI: 1.01–1.04, *P* = 0.007), Procalcitonin (PCT) pod3 (ng/mL) (OR = 1.503, 95% CI: 1.24–11.95, *P* < 0.001), Neutrophil-to-Lymphocyte Ratio (NLR) pod3 (OR = .131, 95% CI: 1.07–1.23, *P* < 0.001), and Platelet-to-Lymphocyte Ratio (PLR) pod3 (OR = 1.012, 95% CI: 1.01–1.02, *P* < 0.001) as independent risk factors. The decision tree prediction model, constructed based on these variables, showed excellent discrimination ability with areas under the ROC curve (AUC) of 0.987 (95% CI: 0.972–1.000) for the training set and 0.990 (95% CI: 0.971–1.000) for the validation set. The calibration curve closely followed the ideal reference line, indicating good model fit. DCA demonstrated that the model had high clinical net benefit across all risk thresholds.

**Conclusion:**

Postoperative day 3 serum inflammatory markers (e.g., CRP, WBC) have high predictive value in identifying early SSI in diabetic patients undergoing single-level PLIF. The prediction model constructed based on these markers performs excellently in terms of accuracy, stability, and clinical utility, making it an effective tool for early identification of high-risk infection patients and providing scientific evidence for individualized postoperative management strategies and interventions.

## Introduction

Surgical site infection (SSI) is one of the most common and serious complications following spinal surgery (1, 2). Posterior lumbar interbody fusion (PLIF), which involves manipulation of deep soft tissue and bony structures, carries a higher risk of postoperative SSI compared to other surgical approaches (3, 4). Previous studies have reported variable incidence rates of SSI after spinal instrumentation, typically ranging from 1.5% to 7.2% (5–8). If an SSI occurs after PLIF, especially in diabetic patients, the clinical manifestations are often atypical and frequently confused with the normal postoperative inflammatory response. “Atypical” refers to a presentation where classic signs of infection—such as high fever, severe incisional pain, or prominent erythema and warmth—may be absent, muted, or delayed. Instead, patients may exhibit only persistent low-grade fever, vague or disproportionate pain, or subtle wound changes like induration or persistent serous drainage. This ambiguity complicates early diagnosis. Delayed treatment may lead to the spread of infection, fixation device infection, and even systemic infection, which can result in functional impairment or even life-threatening conditions (9–11). SSI not only extends hospitalization time and increases medical costs but also delays functional recovery. In severe cases, it may require reoperation, significantly affecting the patient's prognosis and quality of life (12). Therefore, early and accurate identification of high-risk populations and the establishment of a scientifically effective predictive model are crucial for enhancing surgical safety and improving patient outcomes.

Diabetes, a common metabolic disorder with a high global incidence, causes a series of pathological changes in patients, such as impaired leukocyte chemotaxis and phagocytosis, microcirculation disturbance, delayed tissue repair, and collagen synthesis disorders, due to prolonged hyperglycemia (13). These changes are widely recognized as major risk factors for postoperative infection and significantly increase the risk of postoperative infections in surgical patients. Epidemiological data show that diabetic patients are 2–3 times more likely to develop SSI compared to non-diabetic patients, and once infection occurs, prognosis is poorer and mortality is higher. Previous studies have shown that diabetic patients are at a significantly higher risk of infection after orthopedic surgeries compared to non-diabetic patients, particularly in spinal fusion surgeries, which are associated with greater trauma, longer surgery times, and more implanted devices (14, 15). However, there is a lack of systematic studies on the early risk factors for SSI after single-level PLIF in diabetic patients.

Previous studies have explored several risk factors for infection after PLIF, such as hemoglobin, white blood cell count, diabetes, surgery duration, age, BMI, serum amyloid A, C-reactive protein, and lymphocytes (6, 16–18). However, research specifically focusing on the diabetic population remains limited, particularly in the context of single-level PLIF, where the surgical trauma is relatively smaller and the infection risk may differ from multi-level surgeries. Further large-scale studies are needed to clarify this. With the development of artificial intelligence and data mining methods, machine learning has become increasingly prevalent in medical predictive modeling. By integrating preoperative clinical characteristics, intraoperative parameters, and postoperative biochemical markers, a scientific, precise, and personalized risk prediction tool can be developed. This tool would assist physicians in the early identification of high-risk patients and the implementation of targeted interventions. Based on this background, this study aims to retrospectively analyze the early serum biomarkers of SSI following single-level PLIF in diabetic patients, explore their independent risk factors, and construct a quantifiable risk prediction model based on key variables. The model's discriminatory ability, calibration performance, and clinical application value will be further assessed. The study results are expected to provide evidence-based support for preoperative risk stratification and postoperative management, enhancing the safety and therapeutic outcomes for diabetic patients undergoing PLIF.

Furthermore, while recent advances have produced robust models for predicting a wide spectrum of postoperative complication (19), there remains a gap in highly specialized tools tailored for specific, high-morbidity events in uniquely vulnerable subpopulations. A “one-size-fits-all” model may lack the granularity required for precise intervention in specific clinical scenarios, such as distinguishing SSI from the normal postoperative inflammatory response in a diabetic patient after spinal fusion. This study, therefore, aims to develop a precise prediction model specifically for early SSI in diabetic patients undergoing single-level PLIF, leveraging tightly timed postoperative serum biomarkers to provide a targeted decision-making aid for spine surgeons.

## Methods

### Study population

This study retrospectively analyzed patients who underwent PLIF in our Hospital, from January 2010 to January 2024. A total of 2,156 diabetic patients were initially included (excluding 327 patients who underwent multi-level or combined surgeries, 135 patients with preoperative infections or immune system diseases, and 114 patients with missing data or lost to follow-up), leaving 1,680 eligible single-level PLIF diabetic patients. Among them, 165 patients developed early SSI, with an infection rate of 9.8%. As this is a retrospective study, the research protocol was approved by the hospital's Ethics Committee (Ethical Approval No: KYLL-2025-1745). and the study was conducted in strict accordance with the Helsinki Declaration. Personal informed consent was waived, and all data were anonymized. To ensure data continuity and integrity, all patients were required to meet the following inclusion criteria:

### Inclusion criteria

1. Diagnosis of diabetes based on the World Health Organization (WHO) 1999 criteria (20) or the American Diabetes Association (ADA) 2023 criteria (21) (Fasting Plasma Glucose ≥7.0 mmol/L or Hemoglobin A1c ≥ 6.5%);
2. First-time undergoing single-level PLIF surgery, with the surgical segment being L2-S1 or any single segment;
3. Had a documented postoperative hospital stay of at least 7 days, ensuring the retrospective availability of complete laboratory data for the first 3 postoperative days (e.g., fasting blood glucose and inflammatory markers on POD3);
4. Complete medical records, including demographic data, comorbidities, surgical-related indicators, and postoperative infection follow-up data.

### Exclusion criteria

1. Multi-level PLIF surgery (e.g., fusion of L3-L4 and L4-L5 simultaneously) or combined spine surgeries (e.g., laminectomy, other procedures besides pedicle screw fixation);
2. Patients with intraoperative cerebrospinal fluid leakage or surgical site contamination, or those who experienced major intraoperative complications such as massive bleeding or cardiopulmonary failure that could affect serum biomarkers;
3. Pre-existing infectious diseases (e.g., urinary tract infections, pneumonia) or immune system diseases (e.g., rheumatoid arthritis, systemic lupus erythematosus);
4. Severe liver and kidney dysfunction (serum albumin < 25 g/L or serum creatinine > 265 μmol/L), malignancies, or hematological disorders;
5. Patients with more than 30% missing data, making it impossible to confirm the infection status.

### Surgical methods

After satisfactory anesthesia, patients were placed in the prone position, and the surgical area was disinfected. A 6–8 cm vertical midline incision was made at the surgical site, exposing the bilateral laminae and facet joints of the responsible segment. Under fluoroscopy, four pedicle screws were implanted at the responsible segment. The lamina was removed for decompression, and a bone graft was placed in the intervertebral space with a fusion cage. A fixed rod was shaped, implanted, and fixed, followed by lateral bone grafting. The wound was irrigated with saline, hemostasis was achieved, and a drain tube was inserted. The wound was closed layer by layer. Antimicrobial prophylaxis, per the hospital protocol during the study period, consisted of a single 1 g intravenous dose of cefazolin administered within 30 min before skin incision, followed by a second 1 g dose at 24 h postoperatively. This regimen was applied uniformly across the cohort as part of the standard care at the time. A closed suction drain was placed subfascially at the end of the procedure. The drain was typically removed on the morning when the total output over the preceding 24 h was less than 50 mL. Postoperative rehabilitation followed a standardized institutional protocol. All patients were fitted with a thoracolumbosacral orthosis (TLSO) to be worn when upright. Mobilization began with sitting at the bedside and standing with assistance within the first 2–3 postoperative days. Supervised, progressive ambulation was initiated once tolerated with the TLSO in place, typically between the second and sixth postoperative week, based on individual comfort and physiotherapist assessment.

### Identification of early surgical site infection

SSIs were identified and classified according to the 2017 Centers for Disease Control and Prevention (CDC) criteria. For this study, “early SSI” was defined as an infection diagnosed within 30 days of the index PLIF procedure.

The diagnosis was established through a combination of clinical, laboratory, and imaging findings:

1. Clinical Evaluation: Diagnosis was initially suspected based on signs including persistent or new-onset fever (>38.0 °C), worsening incisional pain beyond the normal postoperative trajectory, erythema, warmth, purulent drainage, or wound dehiscence.
2. Laboratory Support: Suspicion was further supported by reviewing serum inflammatory markers (CRP, WBC). A secondary rise or a failure to decline after the initial postoperative peak, in conjunction with clinical signs, prompted further investigation.
3. Imaging Confirmation (for suspected deep SSI): When deep SSI (involving fascial/muscle layers or the intervertebral space) was suspected, advanced imaging—primarily magnetic resonance imaging (MRI)—was obtained. MRI findings consistent with infection (e.g., T2 hyperintensity, T1 hypointensity, fluid collections with peripheral enhancement on contrast-enhanced sequences) were considered confirmatory. Superficial SSIs, involving only the skin and subcutaneous tissue, were diagnosed based on clinical examination findings without the necessity for advanced imaging (22).

### Data collection

#### Basic information

Gender (Male/Female), Age (years), Body Mass Index (BMI, kg/m²), BMI Category (Normal, Obese, Overweight, Underweight), Duration of Diabetes (months), Surgery Duration, Intraoperative Blood Loss (mL), Infection Type (Deep, Shallow).

#### Serum data

HbA1c (%), FPG pre (mmol/L), FPG pod1 (mmol/L), FPG pod2 (mmol/L), FPG pod3 (mmol/L), CRP pre (mg/L), CRP pod3 (mg/L), WBC pre (×10^9^/L), WBC pod3 (×10^9^/L), Albumin pre (g/L), ESR pre (mm/h), ESR pod3 (mm/h), PCT pre (ng/mL), PCT pod3 (ng/mL), NLR pre, NLR pod3, PLR pre, PLR pod3.

### Statistical methods

Statistical analyses were performed using R 4.2.3 software. First, normality tests were conducted for continuous variables. Data that followed a normal distribution were presented as mean ± standard deviation (x̅ ± s), and group comparisons were performed using independent sample *t*-tests. Data that did not follow a normal distribution were presented as median (interquartile range) [M(P25, P75)], and group comparisons were performed using the Mann–Whitney *U-*test. Categorical variables were presented as frequencies (%), and group comparisons were performed using chi-square tests or Fisher's exact test. To identify potential risk factors for early postoperative SSI and avoid the bias of pre-selection, we directly entered all candidate variables collected in this study (as listed in Table 1) into the Least Absolute Shrinkage and Selection Operator (LASSO) regression for variable selection and dimensionality reduction. We selected LASSO for its well-established properties in high-dimensional data settings: it enhances prediction accuracy by mitigating overfitting through L1-penalization and produces a parsimonious, interpretable model by shrinking the coefficients of non-informative variables to zero. While we acknowledge that newer methods (e.g., the knockoff filter) offer enhanced control of the false discovery rate for pure variable inference, the primary goal of our study was to construct a clinically applicable prediction model. LASSO remains a cornerstone technique in clinical prediction modeling for effectively balancing predictive performance, model simplicity, and clinical interpretability. The optimal penalty parameter *λ* was determined by 10-fold cross-validation. Variables selected by LASSO were included in multivariate logistic regression analysis. The data were randomly divided into training and validation sets in a 7:3 ratio. Based on the training set, a decision tree prediction model was constructed. The model's discriminative ability was assessed by plotting the receiver operating characteristic (ROC) curve and calculating the area under the curve (AUC). The goodness of fit and prediction accuracy of the model were evaluated using the Hosmer–Lemeshow test and calibration curves. Decision curve analysis (DCA) was used to assess the clinical net benefit of the model at different probability thresholds, determining its clinical utility. All statistical tests were two-tailed, and a *P* value < 0.05 was considered statistically significant.

**Table 1:** Univariate comparison of patients with early postoperative incision infection and those without infection (*n* = 1,680).

General information	SSI Group (*n* = 165)	Uninfected group (*n* = 1,515)	*P*
Age (years)	64.00 (53.00,74.00)	63.00 (51.00,74.00)	0.338^a^
Gender (*n*%)			
Female	65 (39.4%)	656 (43.3%)	0.379^b^
Male	100 (60.6%)	859 (56.7%)	
BMI (kg/m^2^)	31.66 (25.11,36.57)	29.60 (24.19,35.66)	0.052^a^
BMI Category (*n*%)			
Normal	29 (17.6%)	329 (21.7%)	0.394^c^
Obese	105 (63.6%)	867 (57.2%)	
Overweight	29 (17.6%)	281 (18.5%)	
Underweight	2 (1.2%)	38 (2.5%)	
Time to drain removal (days)	3.0 (3.0, 4.0)	3.0 (3.0, 4.0)	0.215^a^
Diabetes duration (month)	16.96 (9.45,25.23)	17.74 (10.03,26.26)	0.526^a^
Infection type (*n*%)			
Deep part	115 (69.7%)	-	<0.001^c^
Shallow part	50 (30.3%)	-	
HbA1c (%)	9.92 (7.69,12.00)	9.71 (7.62,11.72)	0.315^a^
FPG pre (mmol/L)	13.73 (8.94,17.65)	13.39 (9.45,17.61)	0.862^a^
FPG pod1 (mmol/L)	15.21 (11.08,19.95)	15.63 (10.77,20.52)	0.542^a^
FPG pod2 (mmol/L)	15.36 (10.74,20.36)	14.67 (10.33,19.69)	0.275^a^
FPG pod3 (mmol/L)	17.18 (12.70,22.90)	14.13 (10.18,18.74)	<0.001^a^
CRP pre (mg/L)	5.60 (2.17,8.49)	6.18 (2.95,9.12)	0.056^a^
CRP pod3 (mg/L)	281.70 (133.93,406.80)	111.17 (57.78,162.53)	<0.001^a^
WBC pre (×10^9^/L)	8.12 (5.44,11.04)	8.23 (5.64,11.02)	0.655^a^
WBC pod3 (×10^9^/L)	41.28 (26.70,55.73)	16.90 (10.76,22.81)	<0.001^a^
Albumin pre (g/L)	36.42 (31.06,40.69)	35.16 (29.70,41.00)	0.365^a^
ESR pre (mm/h)	26.00 (15.00,35.00)	25.00 (14.00,36.00)	0.965^a^
ESR pod3 (mm/h)	163.08 (87.07,247.38)	75.00 (43.00,108.00)	<0.001^a^
PCT pre (ng/mL)	0.13 (0.07,0.19)	0.13 (0.07,0.18)	0.809^a^
PCT pod3 (ng/mL)	14.21 (7.83,20.81)	5.79 (3.07,8.94)	<0.001^a^
NLR pre	4.78 (3.15,6.77)	4.75 (2.77,6.93)	0.700^a^
NLR pod3	39.09 (17.64,53.20)	14.68 (8.58,20.71)	<0.001^a^
PLR pre	171.00 (107.00,253.00)	183.00 (110.00,253.00)	0.850^a^
PLR pod3	860.25 (557.19, 1,246.80)	369.00 (226.00,505.00)	<0.001^a^
Surgery duration (min)	146.00 (130.00,157.00)	145.00 (132.00,158.00)	0.536^a^
Blood loss (mL)	205.00 (176.00,228.00)	200.00 (174.00,225.00)	0.361^a^

^a^ Mann–Whitney U.

^b^ Chi-square (*χ*^2^) test.

^c^ Fisher Exact.

## Results

### Baseline characteristics of the total population, training Set, and validation Set

**Figure 1:**
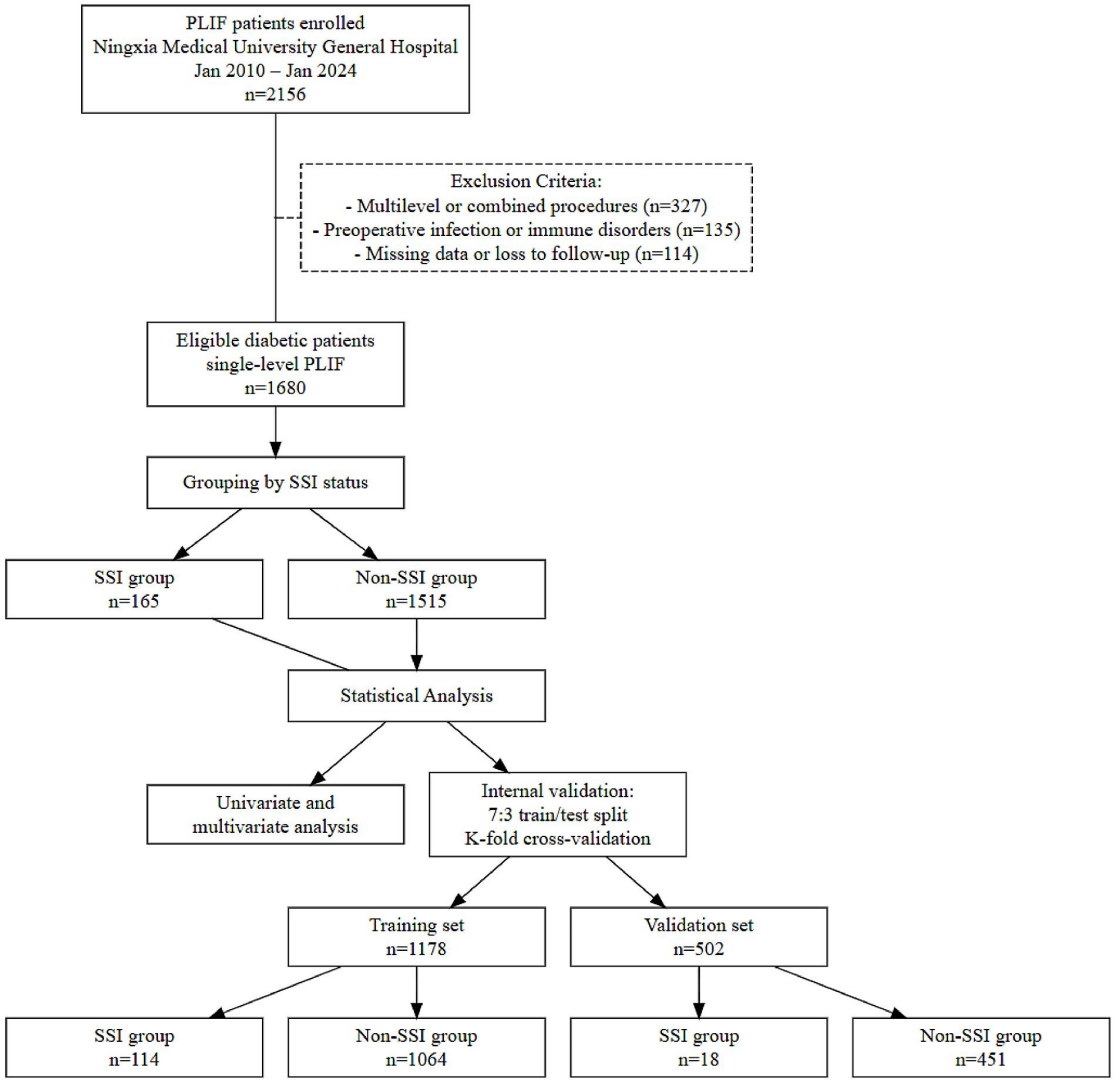
Flowchart of patient screening and grouping.

The patient screening and grouping process in this study is shown in Figure 1. Univariate analysis of early surgical site infection (SSI) after single-level PLIF in 1,680 diabetic patients revealed significant differences between the infection group (*n* = 165) and the non-infection group (*n* = 1,515) in multiple postoperative indicators (Table 1). The fasting plasma glucose (FPG) on postoperative day 3 (FPG pod3) in the infection group was 17.18 (12.70, 22.90) mmol/L, significantly higher than that of the non-infection group, which was 14.13 (10.18, 18.74) mmol/L (*P* < 0.001). Inflammatory markers such as CRP, WBC, ESR pod3, PCT pod3, NLR pod3, and PLR pod3 were all significantly elevated in the infection group (*P* < 0.001).

The data were split into a training set (*n* = 1,178) and a validation set (*n* = 502) at a 7:3 ratio, and baseline characteristics between the two groups showed good balance (Table 2). No significant differences were found between the groups in terms of age, gender, BMI and its categories, HbA1c, and preoperative and postoperative laboratory markers (*P* > 0.05). Only the duration of diabetes showed a borderline difference, with the training set having 16.70 (9.62, 25.69) months and the validation set having 19.70 (10.41, 27.11) months (*P* = 0.028), though the clinical significance was limited. The infection rates in the training set and validation set were 9.7% and 10.2%, respectively (*P* = 0.723), consistent with the overall infection rate (9.8%), and the distribution of deep and superficial infections was balanced (*P* = 0.913). This indicates that the data splitting was reasonable and appropriate for model construction and validation.

**Table 2:** The total number of patients with early incision infection after PLIF, as well as the baseline clinical characteristics of the training set and validation set populations.

General information	Total number (*n* = 1,680)	Training set (*n* = 1,178)	Validation set (*n* = 502)	*χ*^2^/H	*P*
Age (years)	63.00 (51.00, 74.00)	63.00 (51.00, 74.00)	63.00 (51.00, 74.00)	0.19	0.910^a^
Gender					
Female	721 (42.9%)	484 (41.1%)	237 (47.2%)	5.39	0.068^b^
Male	959 (57.1%)	694 (58.9%)	265 (52.8%)		
BMI (kg/m^2^)	29.81 (24.30, 35.75)	29.87 (24.50, 35.78)	29.59 (23.76, 35.67)	0.95	0.622^a^
BMI Category					
Normal	358 (21.3%)	240 (20.4%)	118 (23.5%)	3.68	0.720^b^
Obese	972 (57.9%)	689 (58.5%)	283 (56.4%)		
Overweight	310 (18.5%)	224 (19.0%)	86 (17.1%)		
Underweight	40 (2.4%)	25 (2.1%)	15 (3.0%)		
Diabetes duration (month)	17.70 (9.92, 26.21)	16.70 (9.62, 25.69)	19.70 (10.41, 27.11)	7.17	0.028^a^
Infected					
Infected	165 (9.8%)	114 (9.7%)	51 (10.2%)	0.65	0.723^b^
Uninfected	1,515 (90.2%)	1,064 (90.3%)	451 (89.8%)		
Infection type					
Deep part	115 (6.8%)	82 (7.0%)	33 (6.6%)	0.98	0.913^b^
Shallow part	50 (3.0%)	32 (2.7%)	18 (3.6%)		
Uninfected	1,515 (90.2%)	1,064 (90.3%)	451 (89.8%)		
HbA1c (%)	9.73 (7.65, 11.75)	9.67 (7.60, 11.71)	9.89 (7.68, 11.85)	0.7	0.705^a^
FPG pre (mmol/L)	13.39 (9.41, 17.63)	13.33 (9.45, 17.74)	13.73 (9.23, 17.45)	0.02	0.989^a^
FPG pod1 (mmol/L)	15.60 (10.82, 20.47)	15.56 (10.68, 20.46)	15.79 (11.28, 20.46)	0.8	0.670^a^
FPG pod2 (mmol/L)	14.71 (10.35, 19.78)	14.82 (10.67, 19.73)	14.23 (9.88, 19.90)	2.15	0.341^a^
FPG pod3 (mmol/L)	14.45 (10.35, 19.02)	14.57 (10.33, 19.03)	14.29 (10.45, 18.99)	0.03	0.986^a^
CRP pre (mg/L)	6.12 (2.85, 9.09)	6.18 (2.93, 9.16)	6.01 (2.76, 8.93)	0.63	0.728^a^
CRP pod3 (mg/L)	117.00 (60.19, 172.46)	118.38 (61.92, 174.66)	113.47 (58.25, 168.92)	1.24	0.538^a^
WBC pre (×10^9^/L)	8.21 (5.63, 11.03)	8.27 (5.63, 11.12)	7.97 (5.62, 10.77)	1.04	0.596^a^
WBC pod3 (×10^9^/L)	18.09 (11.27, 23.89)	17.89 (11.21, 23.92)	18.46 (11.63, 23.62)	0.25	0.881^a^
Albumin pre (g/L)	35.31 (29.84, 40.95)	34.99 (29.73, 41.00)	35.94 (30.18, 40.85)	1.25	0.536^a^
ESR pre (mm/h)	25.00 (14.00, 36.00)	25.00 (14.00, 36.00)	25.00 (14.00, 35.00)	0.23	0.893^a^
ESR pod3 (mm/h)	79.00 (46.00, 114.00)	78.00 (44.00, 111.00)	84.50 (50.25, 117.00)	4.36	0.113^a^
PCT pre (ng/mL)	0.13 (0.07, 0.18)	0.13 (0.07, 0.19)	0.13 (0.07, 0.18)	0.09	0.958^a^
PCT pod3 (ng/mL)	6.15 (3.21, 9.51)	6.28 (3.31, 9.57)	5.81 (2.97, 9.34)	1.02	0.600^a^
NLR pre	4.76 (2.78, 6.91)	4.62 (2.68, 6.89)	4.99 (3.16, 6.95)	3.5	0.173^a^
NLR pod3	15.54 (9.02, 22.13)	15.49 (9.07, 21.76)	15.78 (8.93, 23.13)	0.5	0.780^a^
PLR pre	182.00 (109.75, 253.00)	182.00 (110.25, 254.00)	182.50 (107.00, 250.00)	0.09	0.955^a^
PLR pod3	391.50 (240.75, 534.00)	387.00 (235.00, 532.00)	404.00 (258.50, 544.25)	0.65	0.722^a^
Surgery duration (min)	145.00 (132.00, 158.00)	146.00 (133.00, 158.00)	143.50 (131.00, 157.00)	4.39	0.112^a^
Blood loss (mL)	201.00 (174.00, 226.00)	200.00 (174.00, 226.00)	202.50 (176.00, 224.00)	0.2	0.904^a^

^a^ Kruskal, Wallis; U.

^b^ Chi, square (χ^2^) test.

### Group comparisons and Variable selection

**Figure 2:**
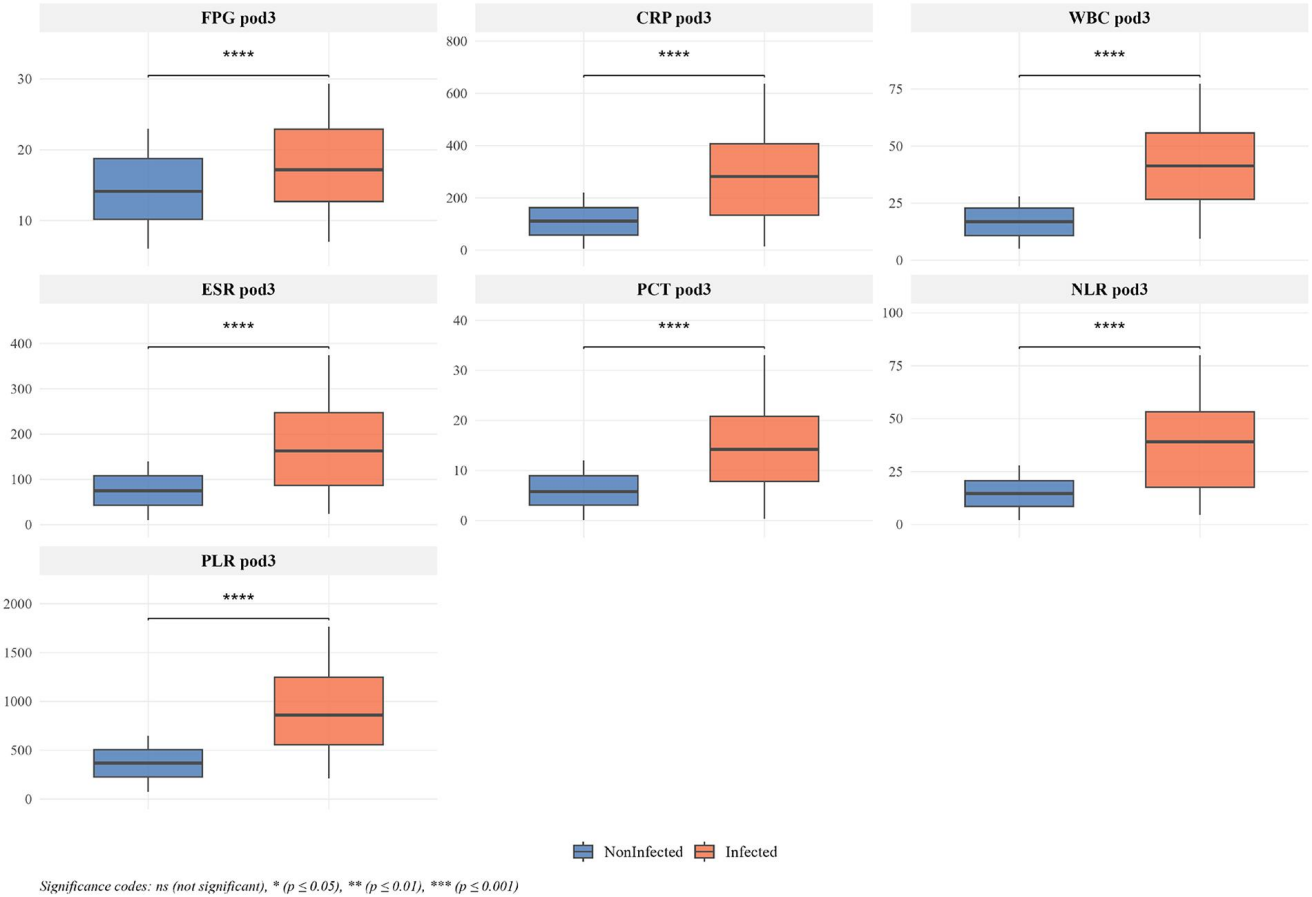
Box plot for univariate analysis of early SSI risk.

**Figure 3:**
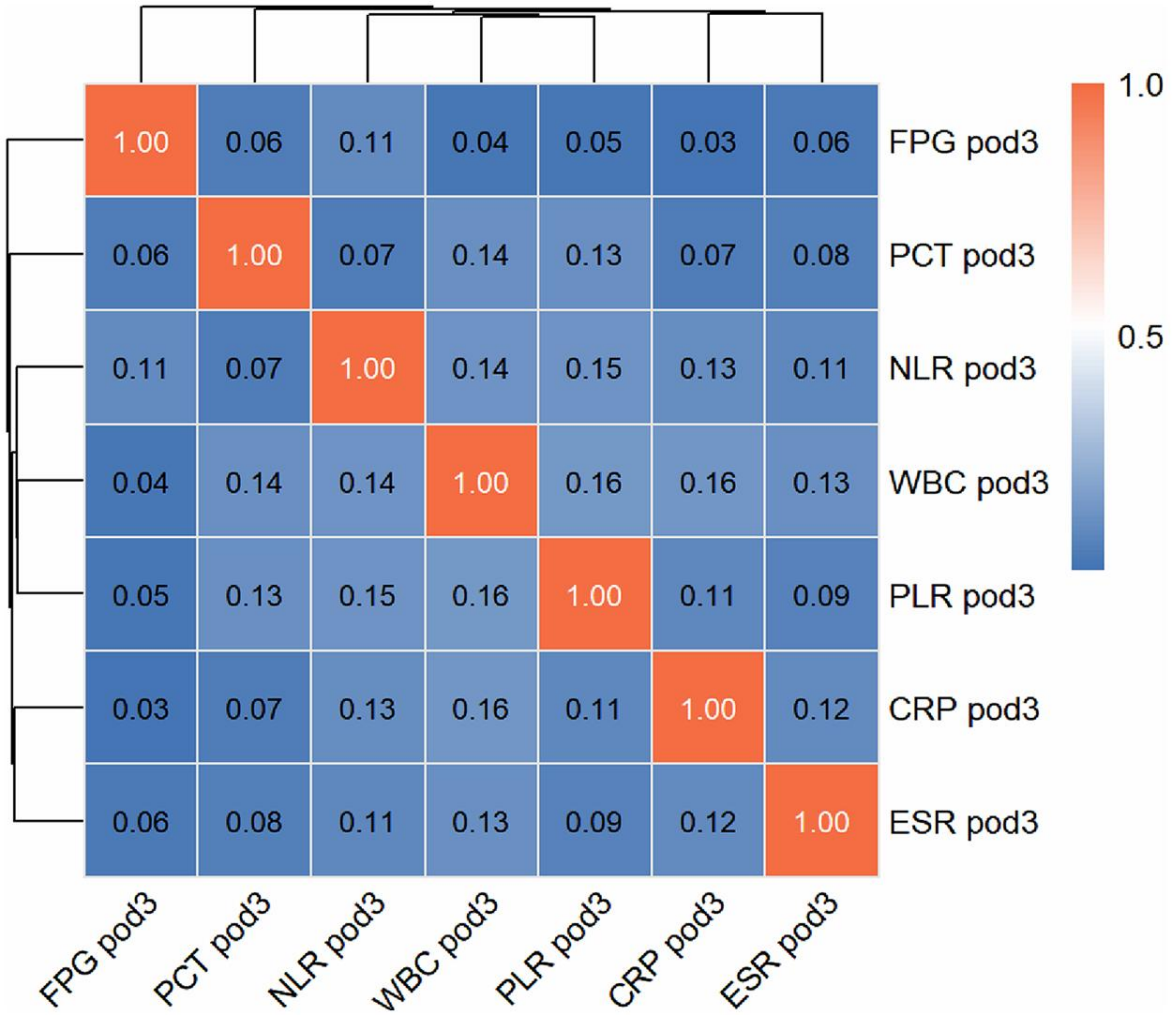
Single-factor analysis of early SSI risk using spearman correlation heat map.

Comparisons between the infection group and the non-infection group revealed significant differences in multiple postoperative indicators, as detailed in Table 1. Given these observed associations, all variables listed in Table 1 were included in the subsequent LASSO regression analysis for formal variable selection, without any pre-filtering based on univariate *P*-values. As shown in Figure 2, the values of each predictive factor were significantly higher in the infection group (the orange boxplots are higher than the blue ones), and the *P*-values for all indicators were less than 0.001 (**** label), indicating significant statistical differences between the infected and non-infected patients. Blood glucose and inflammation-related markers were higher in infected patients. The correlation heatmap in Figure 3 shows that FPG pod3 had a low correlation with other indicators (most correlation coefficients were below 0.1). WBC pod3 had notable correlations with PLR pod3 (0.16) and CRP pod3 (0.16), while PLR pod3 was correlated with WBC pod3 (0.16) and NLR pod3 (0.15). Each indicator was highly correlated with itself (diagonal orange squares), and overall, there were weak-to-moderate correlations between postoperative inflammatory markers, reflecting their potential for synergistic changes during infection, while each also had the potential to independently reflect infection status.

### Variable selection based on LASSO regression

**Figure 4:**
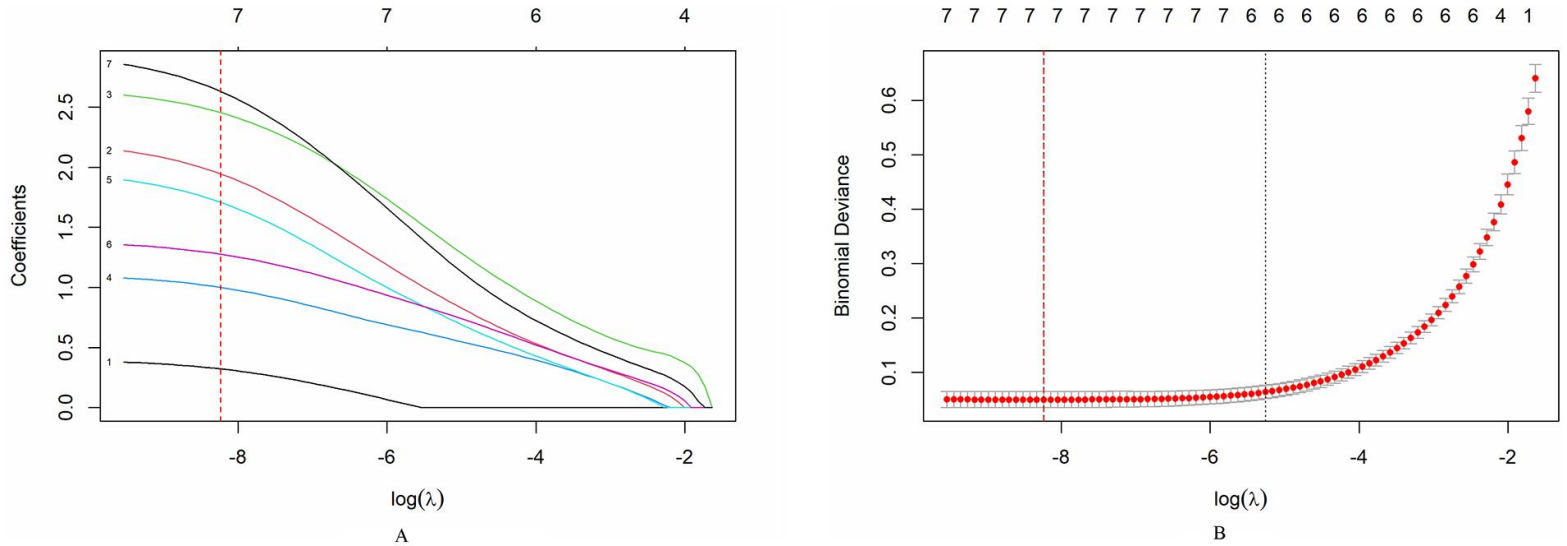
Use LASSO regression to select predictors. **(A)** LASSO coefficient path diagram, drawing the coefficient path diagrams of the 7 predictors based on the logarithmic (*λ*) sequence; **(B)** In the LASSO model, the selection of the optimal parameters generated seven non-zero coefficients at the optimal parameter *λ* (λ = 0.000265).

Using LASSO regression to select features among the seven statistically significant variables from univariate analysis, we determined the optimal *λ* value to be 0.000265, with log(*λ*) = −8 (red dashed line in the figure). At this point, the model selected seven variables (the curve had not shrunk to zero). As log (*λ*) increased (increasing regularization strength), the coefficients gradually shrank, and some variables had their coefficients reduced to zero. This suggests that the chosen *λ* value effectively selected key variables influencing the outcome, providing the basis for constructing a simple and predictive model (Figure 4).

### Collinearity diagnosis

The seven variables selected by LASSO regression were further assessed for multicollinearity by calculating variance inflation factors (VIF) and tolerance values. A VIF >5 or tolerance <0.2 indicates the presence of multicollinearity. As shown in Table 3, the variance inflation factors (VIF) for postoperative inflammatory markers (such as FPG pod3, CRP pod3, WBC pod3, etc.) were all <1.5, and tolerance values were >0.6, indicating no significant multicollinearity. Therefore, these variables were suitable for inclusion in multivariate logistic regression analysis.

**Table 3:** Multicollinearity diagnosis among independent variables.

Variable	VIF	Tolerance
FPG pod3	1.039	0.962
CRP pod3	1.275	0.784
WBC pod3	1.485	0.673
ESR pod3	1.271	0.787
PCT pod3	1.238	0.808
NLR pod3	1.339	0.747
PLR pod3	1.366	0.732

Tolerance: the degree of forbearance; VIF, Variance Inflation Factor.

### Multivariate logistic regression analysis

**Figure 5:**
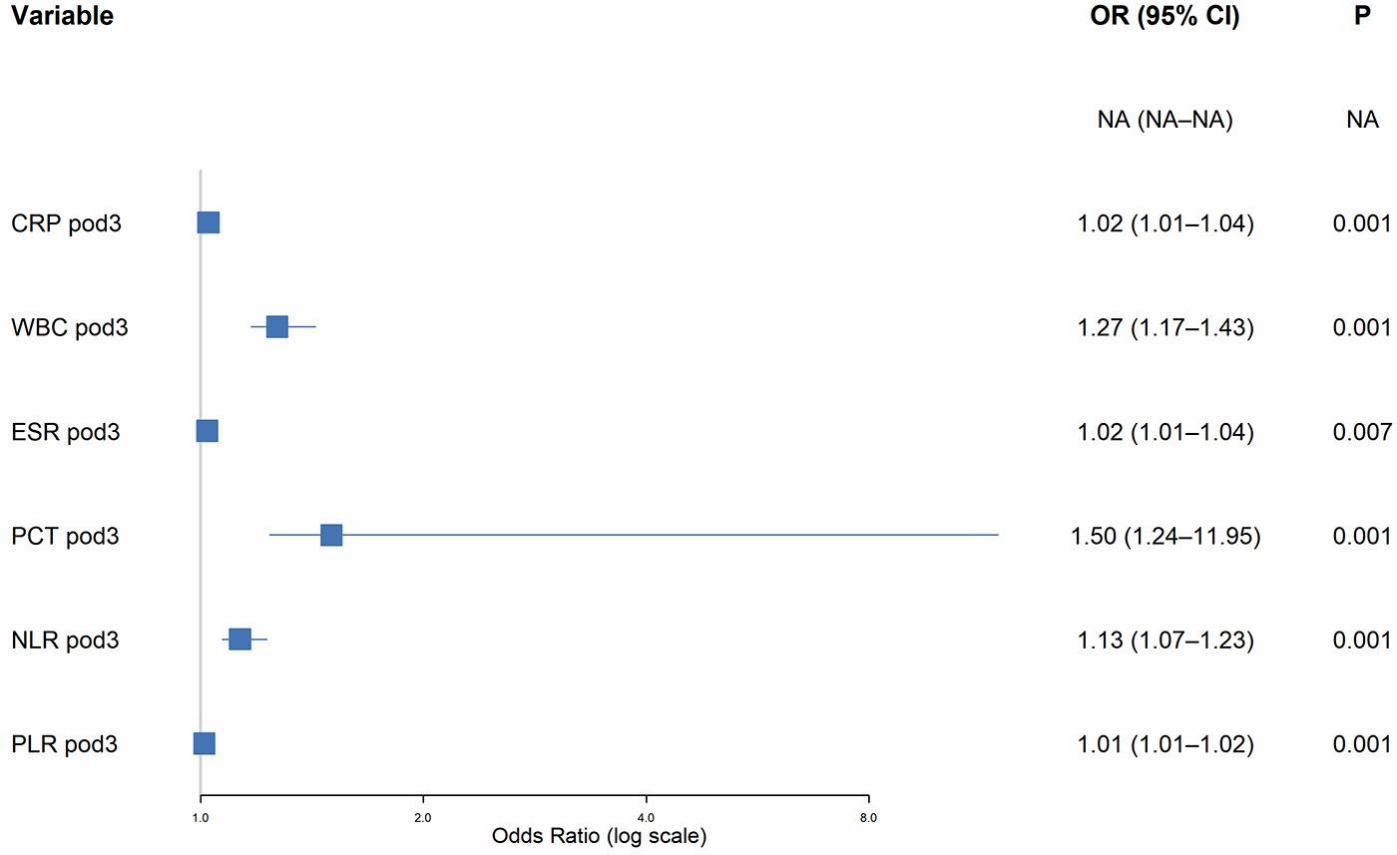
Forest map of independent risk factors for SSI. The horizontal axis represents the logarithmic scale of the odds ratio (OR), and the vertical axis shows the names of each risk factor, sorted in descending order of OR values. Each rectangular box represents an OR value, and its width is proportional to the sample size. The horizontal line represents the 95% confidence interval (CI), and the vertical dotted line corresponds to OR = 1.0 (no risk effect).

The seven variables that passed the collinearity check were included in a multivariate logistic regression analysis, using stepwise backward regression based on AIC to identify the final diagnostic model. The results showed that the final diagnostic model included the following six variables: CRP pod3 (OR = 1.025, 95% CI 1.01, 1.04), WBC pod3 (OR = 1.27, 95% CI 1.17, 1.43), ESR pod3 (OR = 1.021, 95% CI 1.01, 1.04), PCT pod3 (OR = 1.503, 95% CI 1.24, 11.95), NLR pod3 (OR = 1.131, 95% CI 1.07, 1.23), and PLR pod3 (OR = 1.012, 95% CI 1.01, 1.02). FPG pod3 showed no significant correlation in the multivariate model (*P* = 0.258), while all other variables were independently associated with early SSI after single-level PLIF in diabetic patients (*P* < 0.05) (see Table 4). The OR values for all significant variables were greater than 1, and their *P*-values were less than 0.05, indicating that elevated levels of these markers increase the risk of early SSI and can serve as important references for clinical identification of high-risk patients (see Figure 5). The diagnostic model equation for early SSI after single-level PLIF in diabetic patients is:

$$\begin{array}{rl} \mathrm{Logit}(p)= & \left(-33.0455)+0.0164\times \mathrm{CRP}\;\mathrm{pod}3(\mathrm{mg}/L\right)\\ & +0.352\times \mathrm{WBC}\;\mathrm{pod}3(\times 10^{9}/L)+0.0197\\ & \times \mathrm{ESR}\;\mathrm{pod}3(\mathrm{mm}/h)+0.7377\times \mathrm{PCT}\;\mathrm{pod}3(\mathrm{ng}/\mathrm{mL})\\ & +0.1112\times \mathrm{NLR}\;\mathrm{pod}3+0.015\times \mathrm{PLR}\;\mathrm{pod}3. \end{array}$$

**Table 4:** Multivariate logistic regression analysis of patients with early incision infection after plif.

Variable	*β*	SE	Z	Wald χ²	OR	95%CI		P
						Lower	Upper	
Intercept	−27.743	4.113	−6.75	45.51	–	–	–	<0.001
FPG pod3	0.08	0.071	1.13	1.28	1.084	0.95	1.26	0.258
CRP pod3	0.024	0.006	3.85	14.85	1.025	1.01	1.04	**<0** **.** **001**
WBC pod3	0.239	0.05	4.81	23.14	1.27	1.17	1.43	**<0**.**001**
ESR pod3	0.02	0.008	2.71	7.33	1.021	1.01	1.04	**0**.**007**
PCT pod3	0.407	0.114	3.58	12.84	1.503	1.24	11.95	**<0**.**001**
NLR pod3	0.123	0.036	3.44	11.83	1.131	1.07	1.23	**<0**.**001**
PLR pod3	0.012	0.002	4.92	1.28	1.012	1.01	1.02	**<0**.**001**

Bold values indicate independent risk factors, P < 0.05.

### Construction of the SSI diagnostic decision tree

**Figure 6:**
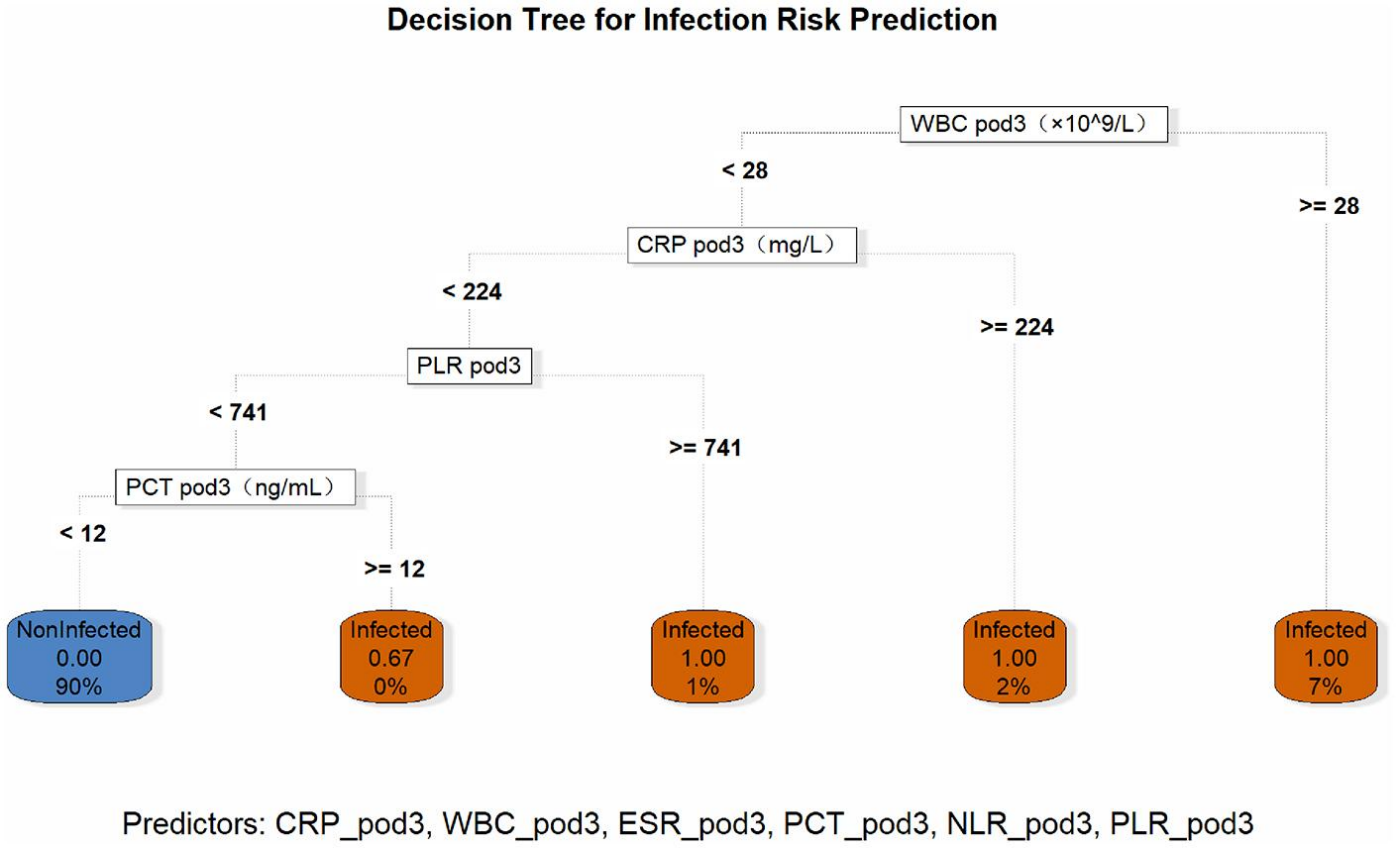
The decision tree prediction model of SSI. The decision tree model constructed based on the training set identifies the key prediction nodes of postoperative infection through the recursive partitioning algorithm. The model depth is 4 layers, including 3 internal decision nodes and 4 leaf nodes, and the overall accuracy rate reaches 92.3%.

The infection risk prediction decision tree was constructed using WBC pod3, CRP pod3, PLR pod3, PCT pod3, and other predictors from the training set. The decision tree established hierarchical rules for infection risk stratification. When WBC pod3 ≥ 28 × 10^9^/L, the infection risk was directly determined. If WBC pod3 <28 × 10^9^/L, further stratification was based on CRP pod3, PLR pod3, and PCT pod3. The leaf nodes of the tree were labeled with infection (Infected) or no infection (Non-Infected), as well as corresponding probabilities and sample proportions, providing a clear and operational decision path for quickly identifying high-risk patients (see Figure 6).

### Model evaluation and validation

#### Model discrimination

**Figure 7:**
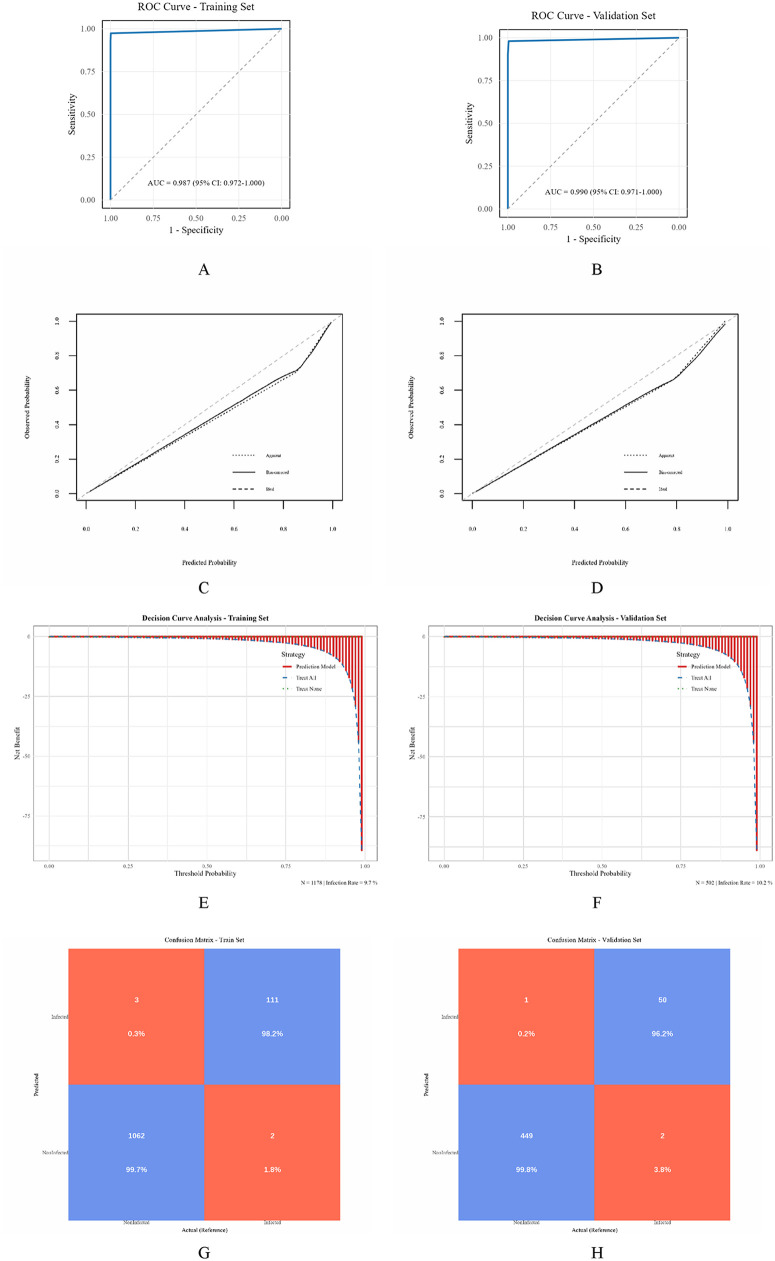
Model evaluation and verification of SSI's decision tree prediction model. **(A)** ROC curve analysis of models in the training set; **(B)** ROC curve analysis of the model in the validation set; **(C)** Calibration curve of the diagnostic model in the training set; **(D)** Verify the calibration curve of the centralized diagnostic model; **(E)** Decision curve analysis of the diagnostic model in the training set; **(F)** Verify the decision curve analysis of the centralized diagnostic model; **(G)** Confusion matrix diagram of the diagnostic model in the training set; **(H)** Verify the confusion matrix diagram of the diagnostic model in the set.

ROC curve analysis showed that the diagnostic model demonstrated excellent discrimination in the training set, with an AUC of 0.987 (95% CI: 0.972–1.000), with the curve close to the top-left corner, indicating strong ability to distinguish between infected and non-infected samples. The model was able to accurately identify high-risk infection patients (see Figure 7A). In the validation set, the model also exhibited good discrimination with an AUC of 0.990 (95% CI: 0.971–1.000), similar to the training set, confirming the model's generalizability and stability in predicting infection risk across different datasets, making it a reliable tool for clinical infection prediction (see Figure 7B).

#### Model calibration

Calibration was assessed using calibration curves and the Hosmer-Lemeshow goodness-of-fit test. As shown in Figures 7C,D, the calibration curves demonstrated a good fit between predicted and observed probabilities. The “Bias-corrected” curves in both the training and validation sets were close to the “Ideal” curves, indicating good consistency between the predicted and actual infection probabilities after correction. Although the “Apparent” curves showed some deviation from the ideal curve, their overall trends reflected the model's predictive ability, with calibration improving accuracy after bias correction. Hosmer-Lemeshow tests in both datasets yielded non-significant results (*P* > 0.05), further indicating good model calibration.

#### Clinical applicability

The clinical applicability of the diagnostic model was evaluated using decision curve analysis (DCA). As shown in Figures 7E,F, the “Prediction Model” curve in the training set showed a higher net benefit than the “Treat All” and “Treat None” strategies when the threshold probability was between 75% and 100%. The trend in the validation set DCA curve was consistent with that in the training set, with better net benefits at higher threshold probabilities. This suggests that using the model to guide infection intervention decisions provides more benefits than blindly treating all or none of the patients, and the model can effectively support clinical decision-making in independent validation data, helping to optimize infection prevention strategies and avoid overtreatment or undertreatment.

#### Confusion matrix

In the training set confusion matrix (Figures 7G,H), the number of correctly predicted infections (Infected) was 111 (98.2%), and the number of correctly predicted non-infections (Non-Infected) was 1,062 (99.7%). Misclassifications were minimal (3 cases predicted as infected but not infected, 2 cases predicted as non-infected but infected), indicating the model's strong ability to distinguish between infection and non-infection in the training data. In the validation set, 50 infections were correctly predicted (96.2%), and 449 non-infections were correctly predicted (99.8%). Misclassifications (1 case predicted as infected but non-infected, 2 cases predicted as non-infected but infected) were rare, showing that the model maintained good classification performance and generalization ability on unseen validation data.

## Discussion

Diabetic patients have a significantly higher risk of surgical site infection (SSI) due to impaired glucose metabolism and immune dysfunction, with the risk being 2–3 times greater than that of non-diabetic individuals (14, 15). PLIF, as a widely used spinal fusion procedure, often leads to serious complications such as epidural abscess, internal fixation loosening, and failure of fixation, which increase the rates of reoperation and mortality (23). Diabetes, surgery duration, and incision size have long been considered risk factors for the occurrence of SSI (7, 24, 25). However, systematic studies on identifying independent risk factors and constructing risk prediction models for early SSI after PLIF in diabetic populations remain scarce. Based on this, the present study evaluates the risk factors for early SSI after single-level PLIF in diabetic patients using postoperative serum indicators and develops an early prediction model, aiming to provide an effective tool for postoperative risk management.

The results of this study indicate that six postoperative inflammation-related indicators, including CRP pod3, WBC pod3, ESR pod3, PCT pod3, NLR pod3, and PLR pod3, were significantly elevated in the infection group, suggesting that the inflammation level on postoperative day 3 is highly correlated with the occurrence of SSI. Among them, CRP and WBC, as traditional markers of infection and inflammation, showed a positive correlation with the risk of infection (for CRP pod3, each 1 mg/L increase increased the infection risk by 2.5%; for WBC pod3, each 1 × 10^9^/L increase increased the risk by 27%), which is consistent with previous studies. H Aono et al. (26) pointed out that CRP, white blood cell count, and body temperature are easily measurable inflammatory markers and can be used as indicators for early SSI after PLIF; Eiichiro Iwata et al. (27) suggested that CRP levels greater than 10 mg/dL on postoperative day 4 help diagnose SSI; Anatoli Pinchuk et al. (28) considered elevated CRP levels as an indicator of increased infection risk. This study further confirmed the predictive efficacy of CRP and WBC in the diabetic population.

Meanwhile, NLR and PLR, as emerging systemic inflammatory scoring markers, have shown good performance in predicting various postoperative complications. In emergency clinics and community-acquired infections, NLR is considered a simpler, faster, and more accurate biomarker for predicting infection compared to white blood cells and CRP (29, 30). Chao-Jun Shen et al. (31) reported that NLR on postoperative day 4 can serve as a prognostic indicator for early SSI after posterior lumbar surgery. A systematic review by Maryam Salimi et al. (32) suggested that NLR may be valuable for early detection of SSI in spinal surgery, and combining it with other markers may improve diagnostic accuracy. Guanglei Zhao et al. (33) indicated that postoperative NLR may have strong predictive ability for early prosthesis-related joint infections. This study also confirmed that NLR and PLR were significantly higher in the infection group compared to the non-infection group, supporting their role as supplementary early warning indicators for SSI risk after PLIF. Furthermore, PCT, a specific marker for bacterial infection, showed superior predictive performance for SSI compared to CRP (in this study, PCT pod3 OR = 1.50), which is highly consistent with the conclusions of Javad Parvizi et al. (34), who found that PCT is more sensitive than CRP and WBC for the early diagnosis of deep SSI, especially in orthopedic implant surgeries.

It is important to note that our model was developed and validated exclusively within a diabetic cohort. Diabetes induces a state of immune dysregulation and chronic inflammation, which may alter the baseline levels, dynamic response, and clinical interpretation of common inflammatory biomarkers compared to non-diabetic individuals. Therefore, the predictive thresholds and variable importance identified here are specific to this high-risk population and may not be directly generalizable. Future studies directly comparing biomarker kinetics and predictive models between diabetic and non-diabetic patients undergoing spinal surgery are warranted to elucidate these differences.

In terms of model construction, this study employed LASSO regression for variable selection to avoid the instability caused by multicollinearity in traditional stepwise regression methods, thus enhancing the robustness of the model. The final model included the six aforementioned variables, and a decision tree model was constructed as a visual tool. The model exhibited excellent discriminative ability in both the training set and validation set (AUCs of 0.987 and 0.990, respectively), with good calibration (Hosmer–Lemeshow *P*-values >0.05 for both). The decision curve analysis (DCA) results showed that the model provided significantly higher net benefit at high-risk thresholds (>75%) compared to “Treat All” or “No Intervention” strategies, demonstrating its strong clinical application value.

Our study deliberately focuses on predicting SSI alone, in contrast to broader models that predict multiple infection types collectivel (19). This focused approach is not a limitation but a strategic choice to enhance clinical utility within a specific context. The pathophysiology and management of SSI, particularly in instrumented spinal surgery, are distinct from those of urinary tract infections or pneumonia. A model that amalgamates these diverse outcomes may optimize for overall accuracy at the expense of specificity for a single, critical complication like SSI. Our specialized model, by concentrating on a homogeneous outcome and population, provides a more targeted risk assessment, potentially offering superior performance and actionable insights for the specific clinical question it is designed to answer.

Previous studies have also attempted to improve SSI risk prediction using machine learning techniques. Tuo Pan et al. (35) applied the XGBoost model to predict early postoperative infections after cardiac surgery, achieving an AUC of 0.96. Beau J Prey et al. (36) used mobile thermal imaging and machine learning techniques to detect early SSI. Q Zhang et al. (37) constructed and validated several models using machine learning, ultimately concluding that the Naïve Bayes model could accurately predict SSI risk with an AUC of 0.95, making it an important tool for early detection and treatment of spinal infections. The model developed in this study achieved a higher AUC in the diabetic population, likely due to the inclusion of more precise postoperative inflammatory indicators and a well-designed modeling strategy. Primoz Kocbek et al. (38) adopted preoperative variable modeling, whereas this study emphasized postoperative inflammation monitoring on day 3, highlighting the value of the “postoperative window period” for early detection.

The strengths of this study include: (1) a large sample size (*n* = 1,680) and the use of training-validation set stratified modeling, which enhances the model's stability and generalizability; (2) a comprehensive prediction tool that incorporates both traditional and emerging inflammatory markers; (3) the use of LASSO and decision tree combined methods, which balances interpretability and predictive accuracy, making the model potentially applicable in clinical settings.

However, there are some limitations. First, as a single-center retrospective study, there may be selection bias, and further validation in multi-center prospective cohorts is needed to assess the model's applicability. Second, this study focused mainly on the postoperative day 3 serum markers and did not consider other potentially important factors, such as preoperative nutritional status and intraoperative factors. Third, this was a single-center retrospective study. Although our sample size is substantial, the generalizability of our findings may be limited by institution-specific practices during the study period. These include the antimicrobial prophylaxis regimen (cefazolin 1 g pre- and 24-hours postoperatively), which does not fully align with current guidelines, as well as our postoperative rehabilitation protocol involving routine bracing and a cautious timeline for ambulation. These contextual factors should be considered when interpreting our results and applying the model in settings with different standards of care. Fourth, this study lacked a non-diabetic control group. A comparative analysis would help clarify whether the identified risk factors and their predictive power are unique to the diabetic state or represent universal markers of SSI risk. External validation in multi-center, prospective cohorts is an essential next step to confirm the model's robustness and clinical utility across diverse healthcare settings before widespread implementation can be recommended. Additionally, regarding model evaluation, our assessment focused on AUC-ROC, calibration, and clinical utility via DCA, with confusion matrix analysis confirming high sensitivity and precision. We acknowledge the recommendation by contemporary methodologies to also report the area under the precision-recall curve (AUC-PR) for a comprehensive assessment of performance on imbalanced datasets. The incorporation of AUC-PR and related metrics (e.g., F1 score) will be a standard component of our analytical framework in all future predictive modeling research to ensure the highest level of methodological rigor and comparability.

Future research could extend to dynamic multi-point data collection, combining preoperative glucose metabolism control, physiological function assessments (e.g., FRAIL score), intraoperative anesthesia and medication details, and radiomics features to construct a multimodal joint prediction model. Additionally, the ability of deep learning and neural network models for data mining could be explored, enhancing automation and personalization while maintaining model performance. The ultimate goal is to achieve real-time identification and intelligent early warning for SSI risk, contributing to the precision development of perioperative management after PLIF surgery. Finally, regarding our methodological approach, we selected variables using LASSO regression due to its strengths in prediction for clinical models. We acknowledge the insightful perspective that advanced techniques, such as the knockoff filter, could provide more rigorous statistical guarantees regarding the control of false discoveries during variable selection. Applying such state-of-the-art methods in future research with larger, multi-center datasets represents a valuable direction to further refine and validate the specific risk factors identified in our current study.

This model identifies diabetic patients at high risk for early SSI following single-level PLIF. In practice, a high predicted probability could serve as an alert to clinicians, prompting earlier and more targeted investigations, such as focused physical examination, serial inflammatory marker trending, or advanced imaging. This may facilitate timely decision-making regarding the need for further diagnostic procedures (e.g., culture acquisition), infectious disease consultation, or consideration of therapeutic interventions, all of which should be tailored to the individual patient's clinical presentation. Future prospective studies are needed to validate the effectiveness of such a risk-stratified management pathway in improving patient outcomes.

## Conclusion

This study provides a new strategy for infection prevention and control in diabetic patients after PLIF surgery. We recommend routine monitoring of postoperative day 3 inflammatory markers and using the model to implement individualized risk stratification for precise intervention. However, as a single-center retrospective study, further multi-center prospective research is needed to verify the model's generalizability and optimize the prediction system by incorporating more indicators.

## Data Availability

The raw data supporting the conclusions of this article will be made available by the authors, without undue reservation.
